# Program on immunization and cold chain monitoring: the status in eight health districts in Cameroon

**DOI:** 10.1186/1756-0500-6-101

**Published:** 2013-03-16

**Authors:** Jérôme Ateudjieu, Bruno Kenfack, Blaise Wakam Nkontchou, Maurice Demanou

**Affiliations:** 1 Department of Biomedical Sciences, University of Dschang, Cameroon, PO Box 067, Dschang, Cameroon; 2 Public Health Laboratory Unit, Regional Hospital of Bafoussam, Ministry of Public Health, PO Box 980, Bafoussam, Cameroon; 3 Arbovirus and Haemorragic Fever Virus laboratory and WHO National Reference Laboratory for yellow fever and measles, Centre Pasteur Cameroon (member of RIIP), PO Box 1274, Yaoundé, Cameroon; 4 Division of Health Operations Research, Ministry of Public Health, Cameroon, PO Box 33490, Yaoundé, Cameroon; 5 Department of Mother and Child, Dschang District Hospital, PO Box 43, Dschang, Cameroon; 6 Cameroon National Expert Committee on Adverse Events Following Immunization, Yaoundé, Cameroon; 7 Department of Epidemiology and Public Health, Swiss Tropical and Public Health Institute, Swiss Tropical and Public Health Institute (associated Institute of the University of Basel), Socin str. 57, P. O. Box, 4002, Basel, Switzerland

**Keywords:** Cold chain, EPI, Monitoring, Health districts, Vaccination campaign

## Abstract

**Background:**

Cold chain monitoring is a precondition to ensure immunization quality, efficacy and safety. In Cameroon, the Expanded Program on Immunization (EPI) has National Standard Operating Procedure (SOP) that describes the vaccines, the cold chain system and equipment, its use and recommended procedures to control and monitor the temperatures and the cold chain. This study was conducted to assess the status of cold chain in eight health districts in Cameroon.

**Findings:**

The study was carried out in eight health districts out of fifty with poor immunization coverage rate. Data were collected using a validated form by observation and consultation of related documents. District Health Services (DHS) and four Integrated. Health Centers (IHC) randomly selected were targeted per health district. Forty health facilities were included. Twenty eight (70.0%) had at least one functional refrigerator for EPI activities. The power supply was reported to be permanent in 7 (20.6%) out of 34. (85.0%) health facilities with access to power supply. The temperature monitoring chart was pasted on 27 (96.4%) of the cold chain equipment. On 16 (59.3%) of these charts, the temperature was recorded twice daily as recommended. Seven (25.9%) of 27 refrigerators assessed had temperature out of the recommended range of 2 to 8°C. Almost 23.30% of health centers did not received any supervision on cold chain monitoring during a vaccination campaign.

**Conclusion:**

This study documents failure of the cold chain maintenance and questions the efficacy and safety of vaccines administered during EPI activities in Cameroun. These findings indicate that appropriate actions are needed to ensure monitoring of EPI cold chain in the country.

## Findings

### Introduction

Immunization is unquestionably one of the most cost-effective public health interventions available [1]. In order to improve its accessibility to children worldwide, the World Health Organization (WHO) launched the Expanded Program on Immunization (EPI) in 1974 with as objective to prevent seven of the most serious diseases [2]. It was introduced in Cameroon since 1976. Its objective has been actualized in the country since 2001 in order to control vaccine preventable diseases among children, reaching by 2015, the DTPw-HB/Hib coverage rate of 90% at national level and 80% in each Health District [3]. Achieving this objective depends on the quality of vaccines used. Given its composition, to preserve its potency and safety, each vaccine should be strictly kept within a specific range of temperature from manufacturer to the recipient.

The maximum vaccine potency is preserved by, among other things, maintaining its functional cold chain system at all levels. It implies for those involved, mastering vaccines sensitivity to temperature and being adequately skilled and equipped regarding conditions of storage and transportation for each vaccine as well as cold chain and power supply monitoring [4-11].

The Cameroon National Standard Operating Procedures (SOPs) for EPI activities is a dynamic document that recommends to store vaccines at different levels as follow [12]:

- Central level, for less than six months;

- Regional Delegations of Public Health, for less than three months;

- District Health Services (DHS), for less than one month;

- Integrated Health Centers (IHC), for less than one month.

It also describes among others: the vaccines, the cold chain system, its use and recommended procedures for temperature monitoring. It is periodically updated and should be available in all health facilities that implement EPI activities. As stated in this document, ranges of temperature in which vaccines are stored depend on the level of the health system and the type of vaccine. Vaccines such as OPV, measles vaccine, BCG can be safely frozen at central, regional and district levels. TT (tetanus toxoid vaccine) and DTPw-HB/Hib should not be frozen. At IHC and in health facilities and during immunization sessions, all vaccines should be stored between +2°C and +8°C. The monitoring of cold chain at each level is to be insured by trained personnel. Each health facility in charge of storing vaccines or organizing vaccination sessions should have adequate functional cold chain equipment. To monitor the temperature in the freezer or refrigerator used to store vaccines, temperature should be read twice daily and recorded on the temperature sheet pasted on it. Temperatures out of recommended range are recorded in red. A plan of contingency to maintain vaccines in recommended range of temperature when the cold chain equipment is broken or when power supply is interrupted should be pasted on cold chain equipment and implemented as indicated. To follow up and monitor EPI activities, all IHC and DHS personnel have to be periodically trained, supervised and evaluated.

Health care delivery in Cameroon has as objective to make Primary Health Care (PHC) accessible to the entire population through the decentralization of the health management process to the health district level [13]. Thus the health system is organized in three levels including the central, the regional and the Health District. The health policy and strategies are elaborated from central level and implemented at the district level by the DHS. Resources are mainly allocated by the state budget, local communities, international and national organizations.

The country is divided in ten Health Regions. Each of these regions is geographically compartmented in health districts. The health district is a geographic area that covers a population of 30,000 to 400,000 inhabitants. It is divided in health areas covering 5,000 to 30,000 inhabitants. In each area, the integrated Health Centre (IHC) is in charge of providing the Minimum Package of Activities (MPA) (smallest set of activities which is identical at all health units of the same level). The implementation of EPI is part of this package. It includes storing vaccine as recommended in the national SOPs, organizing and reporting vaccination sessions, conducting epidemiological surveillance of disease targeted by EPI. It targets with 10 vaccines, all children under one year, pregnant women and other specific groups during routine EPI and supplementary vaccination activities.

Power supply in the Cameroon varies from one locality to another [14]. Hydroelectricity is the cheapest and main power source in urban and semi urban areas, but it is still not available in many rural localities where kerosene, solar, natural gas and in some occasion generators are the main sources for cold chain. Considering the unequal distribution of power supply, the lack of information on availability of cold chain equipment, the shortage of trained and motivated health personnel, this study was designed to attempt to answer to the question whether in targeted health districts, cold chain status at health district level complies with the national SOPs.

### Methods

**Figure 1:**
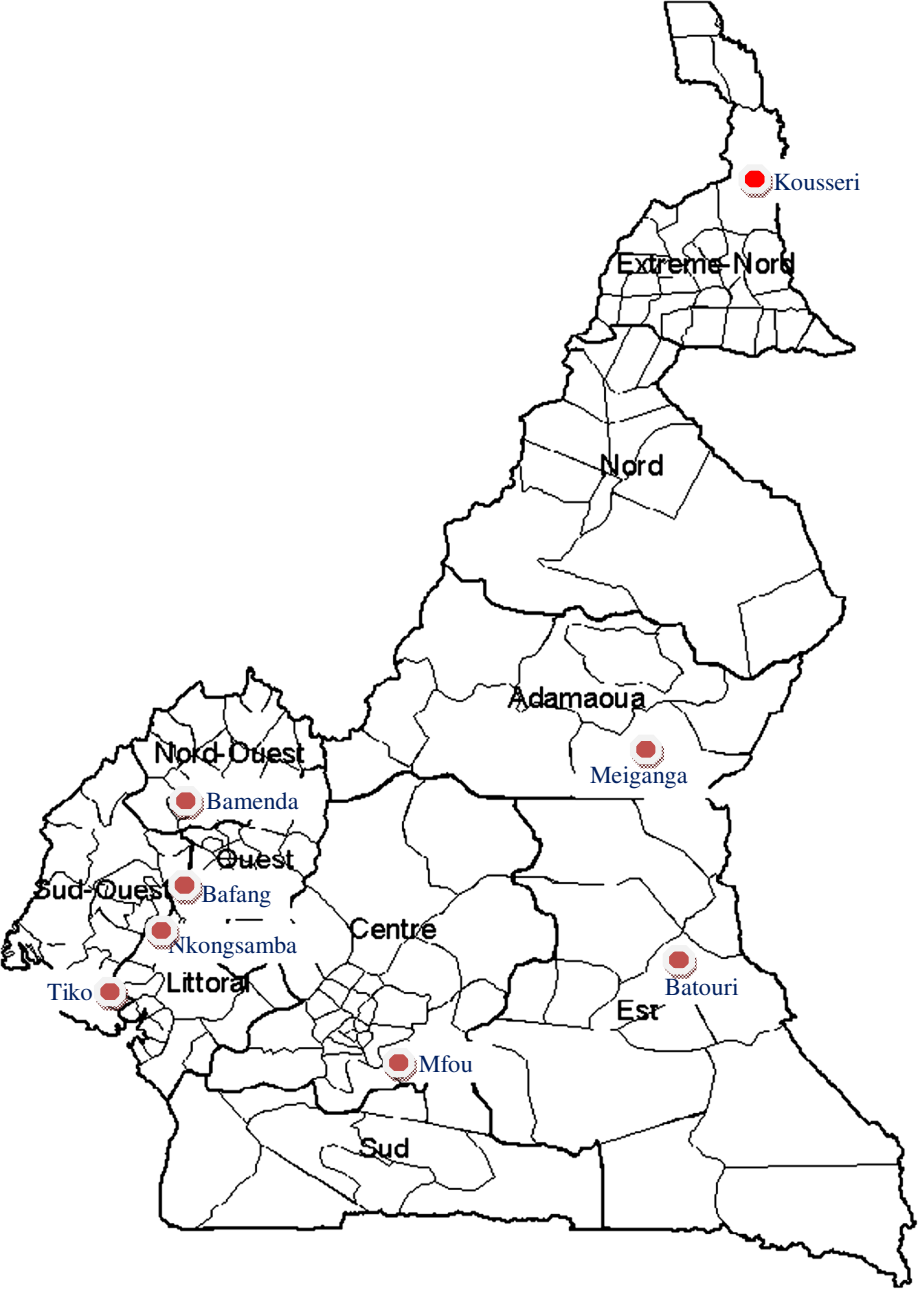
Map of Cameroon showing the selected health districts within different Regions.

It was a cross sectional study, conducted in eight health districts of Cameroon (Figure 1). These included: Kousseri (Far North Region), Meiganga (Adamawa Region), Batouri (East Region), Nkongsamba (Littoral Region), Tiko (South West Region), Bamenda (North West Region), Bafang (West Region) and Mfou (Center Region). This study was part of the evaluation of the national mass vaccination campaign implemented in December 2008 and targeting pregnant women and children under five. It was ordered by the Cameroon Ministry of Health to identify factors associated to low immunization coverage rate.

Our sampling frame consisted of Health Districts needing a campaign to improve its 2008 annual immunization coverage among children and pregnant women. These included 50 health districts with the lowest performances in terms of TT coverage among pregnant women during the first half of the year and were selected from 8 of the 10 Health Regions of the country (Figure 1).

In each Health Region, Health Districts were ranked according to the coverage rate of the second dose of TT among pregnant women, and the district with the lowest coverage rate was selected. In each health district the DHS and four IHC randomly selected were included.

The data collection tool was developed from the cold chain supervision grid in the SOPs on EPI in Cameroon. It was pre-tested in the Biyem Assi DHS (in the Centre Health Region) and in four IHC of the same district, reviewed and the last version adopted by authors. All surveyors were health personnel who had been previously involved in EPI activities at district level and were trained and evaluated before their involvement in the data collection process.

Data were collected by observation of the cold chain and by consulting related documents.

This was done on the availability of cold chain equipment, power supply, ice packs, temperature record sheets and thermometer. Information on vaccines and diluents storage conditions, temperature recording and its variations out of recommended ranges in two previous months and at the moment of evaluation was also collected. In addition, it was verified and recorded if ice packs were adequately disposed and if food or other non recommended products were found in the refrigerator. Collected data were coded and entered in Excel 2007 then transferred and analyzed in Epi Info version 3.5.3. As there were no intervention or interaction with human beings or any of their data, no informed consent or ethical clearance were necessary. However, authorization to conduct this study in the various health districts of the country was granted by the Minister of Health and the head of the health institutions.

### Results

Forty health facilities including eight DHS and 32 IHC were involved in this study. These were from 8 health districts with 7 covering urban and rural areas, whereas one covered only a rural area. Twenty five (78.1%) of IHC were located in rural areas, whereas 7 (21.9%) were in urban areas. The estimated total population size of included health districts ranged in 2008 between 79,100 (Mfou Health District) to 269,200 inhabitants (Bamenda Health district).

Twenty nine (72.5%) out of 40 health facilities had at least one refrigerator (Front-loading refrigerator with freezer on top or Ice Lined Refrigerator). Twenty one (52.5%) of them had at least one freezer. The availability of functional refrigerators and freezers is indicated in Table 1.

**Table 1:** Observed conditions of the cold chain status in health facilities (n = 40, including 8 districts health services (DHS) and 32 integrated health centers (IHC)) in Cameroon, December 2008

**Observed conditions of the cold chain status in health facilities**	**Number of health facilities (%)**			
	**Total**	**DHS**	**IHC**	P value*
Availability of at least one functional refrigerator in health facilities	28 (70.0)	8 (100.0)	20 (62.5)	0.0404
Availability of at least one functional freezer in health facilities	9 (22.5)	6 (75.0)	3(9.4)	0.0005
Access to power supply				
At least one source	34 (85.0)	8 (100.0)	26 (81.3)	0,2361
The power simply was permanent (total with power supply =34)	7 (20.6)	0 (0.0)	7 (26.9)	0.0900
Sources of power supply				
Electricity as main source	21(61.8)	8 (100.0)	13 (50.0)	0,0112
Gas as main source	12 (35.3)	0 (0.0)	12 (46.2)	0.0176
Kerosene as main source	01(2.9)	0 (0.0)	01(3.8)	0.7647
Temperature recording				
Temperature recording sheets pasted on refrigerators (total with refrigerators = 28)	27 (96.4)	8 (100.0)	19 (95.0)	0.0900
Thermometer available in refrigerators	27 (96.4)	7(87.5)	20(100.0)	0.1071
Temperature properly recorded (complete ad update)	16 (40.7)	5(71.4)	11 (55.0)	0.0872
Refrigerators with temperature within the recommended range (2-8°C) at the moment of observation	20 (70.1)	5 (71.4)	15(75.0)	0.3321
Proper storage of vaccines in the refrigerator	22 (78.6)	5 (62..5)	17 (85.5)	0.0392
Proper parking of ice packs in refrigerators	13 (46.4)	2 (25.0)	11 (55.0)	0.0314
Refrigerators with vaccine vial monitor that has change	3 (10.7)	1 (12.5)	2 (10.0)	0.1146
Refrigerators with other product than vaccine, diluents and ice packs	8 (28.6)	4 (50.0)	4 (20.0)	0.0235

* P value obtained from Fisher Exact Test.

At the moment of evaluation, access to power supply was reported in 34 (85.0%) health facilities. The distribution of sources of power supply in health facilities is indicated in Table 1. The power supply was reported to be permanent in only 7 (20.6%) of health facilities.

Table 1 also presents the availability of thermometer and temperature monitoring chart pasted on cold chain equipment. It was noted that the temperature was not systematically recorded on charts twice daily as required on the chart in 11 (40.7%) of the 27 health facilities with temperature monitoring charts on refrigerator. During the period of two months preceding the study, only 16 (59.3%) of these 27 health facilities, recorded daily temperatures within the recommended range. For the same period, 11 refrigerators (40.7%) recorded 72 times the temperatures out of the recommended range. Actions taken after theses failures were not documented where indicated. At the moment of the study, up to 7 (25.9%) of the 27 refrigerators assessed had temperatures out of the recommended range. The proportion of health facilities with temperatures within the recommended range, proper storage of vaccine and ice packs, vaccine vial monitor that has changed and with no recommended product are presented in Table 1. It was noted that up to 6 (20.69%) health facilities had wrong packing of vaccines and diluents in the refrigerator. These included: not arranging vaccines so as to facilitate air circulation and reading of their identification, as well as expired date; not marking and arranging separately vaccines brought back from immunization session; and not storing vaccines in locations appropriate to the style of refrigerator used (e.g.: for ILR refrigerator, storing adsorbed vaccines (DTP, TT, HepB) on the top, OPV and freeze dried vaccines (measles, BCG) on the bottom). In 4 (13.79%) of the health facilities there were some vaccines without label in the refrigerator. Nevertheless, no vaccine with an expired date was found. It was noted that up to 23.30% of health centers did not received any supervision during the vaccination campaign that preceded this evaluation.

### Discussion

The purpose of this study was to evaluate whether the monitoring of cold chain for national

EPI program in targeted health districts complied with the SOPs. Almost 27.50% of the health facilities were conducting EPI activities without any cold chain equipment. In addition, access to power source was not permanent; cold chain required material such as thermometer and temperature monitoring charts was lacking in some health facilities. Activities that are required to ensure cold chain for vaccines such as correct parking of vaccines and ice packs in the cold chain equipment, recording temperature twice a day were not systematically implemented.

The absence of cold chain equipment documented in more than a quarter of health facilities insuring EPI activities was a major preoccupation since these health facilities are obliged to store their vaccines in other health facility and to travel before and after immunization sessions to collect and deposit vaccines. This increases the risk of exposing vaccine to heat temperatures and can seriously hamper the organization of immunization sessions, especially in outreach and mobile strategies. It can also lead to increasing vaccines wastage rates and stock outs. This weakness had been documented in Bongor (Chad) in 1994 and was associated with increase of incidence of measles cases among vaccinated children [11]. The situation is expected to be worse in Cameroon if we combine this limited access to cold chain equipment to that of power supply (in 15% of health facilities visited in this study) and intermittent power supply (in 79.4% of health facilities with power supply in the present study). Irregular power supply of health facilities and absence of standby generator were identified in a previous study conducted in Nigeria as one of major risk factors of lose of vaccine potency [15].

It was a positive point to note that for 93% of cold chain equipment, temperature monitoring charts, thermometer, and ice packs were available. This indicates a certain degree of implementation of cold chain monitoring following the national and international EPI Standard Operating Procedures [13,16,17]. But actions are needed to ensure day to day monitoring of cold chain to improve the actual situation where for 40.7% of cold chain equipment in two months, the temperature was not recorded twice daily as recommended. This situation makes unavailable the information on vaccine exposure to temperatures out of recommended ranges as well as wrong parking of vaccines (21.4% of health facilities) and ice packs (53.6% of health facilities). The insufficient supervision coverage (23.30% of health facilities) illustrates failure to implement Standard Operating Procedures for EPI in a considerable proportion of health facilities in Cameroon. Although not investigated during this study it can be explained by absence or insufficient training, supervision, motivation, access to guideline or work load due to lack of health personnel. Similar situation was recently documented in Ethiopia [4] where 29.7% and 12.5% had respectively incomplete and absence of temperature recording on temperature monitoring charts.

Various weaknesses, documented from this study, have been underlined from other studies as major risk factors associated with exposure of vaccine to temperature out of recommended ranges, lose of vaccine potency, low immunization coverage rate, increase incidence of cases of disease despites the vaccines [16-18]. Some key interventions including constant supervision, training of professionals in charge, promoting access to existing guidelines and making available cold chain tools and equipment in health facilities have been identified to reduce theses gaps [19-21]. Furthermore, innovating strategies like computerizing temperature monitoring of the vaccine cold chain and developing thermostable vaccines are being tested and could improve the protection of vaccine potency in developing countries context [22-24].

Included health districts were not randomly selected and this predisposed to selection bias if we were to generalize these results to the whole Cameroon. Data were mainly collected from documents by specialized and supervised trained surveyors with a validated standardized form. Thus these results are less likely to be explained by any measurement bias due to the data collection process. Despite these limitations, this study succeeded in picturing vaccines cold chain status and needs of actions to reduce identified gaps.

### Conclusion

Although, we cannot infer the results of this study to all Cameroon health districts, its findings described weaknesses in cold chain maintenance in the country and pulling the communication cord is therefore needed for action in the whole country. The failure of the cold chain maintenance documented here questions the efficacy and safety of vaccines that are administered during EPI vaccination sessions in Cameroun. This can have as consequences, lose of potency of administered vaccines, low immunization coverage rate, increasing incidence of cases of disease and adverse events following immunization among vaccinated persons. To improve this situation in Cameroon, we recommend:

1. To the Cameroun Ministry of Health

a. To map access to power supply in health facilities in charge of implementing EPI activities and provide those with no access to hydroelectricity with alternative sources of power like solar, kerosene and gas and adapted refrigerators and freezers.

b. Insure that all health facilities involved in EPI activities have adequate cold chain equipment (refrigerator and or freezer).

c. Provide health facilities with access to hydroelectricity with secondary sources of power.

d. Insure that all health facilities implementing EPI activities have the Standard Operating Procedure for EPI in Cameroon.

2. To District and regional health authorities:

a. Insure that refrigerators and freezer used to stored vaccines are equipped with all recommended tools,

b. Organize to prevent power interruption in health facilities by stocking alternative sources of power like solar, gas and kerosene,

c. Appoint in all health facilities, health personnel for cold chain monitoring and evaluate, train and supervise them at a reasonable periodicity,

d. Identify and address factors leading to failure of cold chain monitoring.

3. Sponsors and researcher should work to manufacture thermostable vaccines, provide all vaccine with Vaccine Vial Monitor and to computerize cold chain temperature monitoring.

## Competing interests

The authors declare that they have no competing interests.

## Authors’ contributions

JA conceived the study. JA, BK, and BWN organized and coordinated collection of field data. JA and MD guided the study design and coordinated the review. JA analyzed data and drafted the manuscript. MD finalized the manuscript. All authors read and approved the final manuscript.

## References

[B1] Ehreth J The global value of vaccination Vaccine 2003 21 596 600 10.1016/S0264-410X(02)00623-0 12531324

[B2] World Health Organization Global programme for vaccines and immunization: expanded programme on immunization. Safe vaccine handling, cold chain and immunizations 1998 Geneva http://www.old.health.gov.il/download/forms/a3039_GDPv.pdf

[B3] Ministry of Public Health Decision n° 0333/MSP/CAB of July 29, 2002 reorganizing the expended program of immunization in Cameroonhttp://acdevcm.free.fr/sante/pev.html23579427

[B4] Berhane Y Demissie M Cold chain status at immunization centres in Ethiopia East Afr Med J 2000 77 476 479 12862137 10.4314/eamj.v77i9.46692

[B5] Gazmararian JA Oster NV Green DC Chuessler L Howell MSK Davis J Krovisky M Waeburton SW Vaccine storage practices in primary care physicians offices Am J Prev Med 2002 23 4 246 253 S 278 10.1016/S0749-3797(02)00512-3 12406478

[B6] Hanjeet K Lye MS Sinniah M Schnur A Evaluation of cold chain monitoring in monitoring in Kelantan, Malaysia Bull World Health Organ 1996 74 391 397 8823961 PMC2486883

[B7] Miller NC Harris MF Are childhood immunization programmes in Australia at risk? Investigation of the cold chain in the Northern Territory Bull World Health Organ 1994 72 3 401 408 8062398 PMC2486708

[B8] Thakker Y Woods S Storage of vaccines in the community: weak 343 link in the cold chain? BMJ 1992 304 756 758 10.1136/bmj.304.6829.756 1571683 PMC1881607

[B9] Bass AG The warm chain: a critical problem for national immunization programs in temperate and colder climates *BASICS* February 1996; manila, Philippines; TechNet. *A basic support for institutionalizing child survival project (BASICS) assignment report* http://www.path.org/publications/files/TS_cc_evidence.pdf

[B10] Boa A Approaches of the expanded programme of immunization (EPI) and analysis of its failures in the sanitary district of Bouna (northeast of Côte d’Ivoire) Bull Soc Pathol Exot 2006 99 5 386 390 17253058

[B11] Luthi JC Kessler W Boelaert M A survey on vaccine efficacy in the city of Bongor (Chad) and its operational consequences for the vaccination program Bull World Health Organ 1997 75 5 427 433 9480198 PMC2487021

[B12] Ministry of Public Health Standard operating procedure for EPI in Cameroon. Expanded program on immunization 2009 37 53 http://minsante-cdnss.cm/content/normes-et-standards-du-programme-elargi-de-vaccination

[B13] Owona Essomba R Bryant M Bodart C The reorientation of primary health care in Cameroon: Rationale, obstacles and constraints Health Policy & Planning 1993 8 3 232 239 10.1093/heapol/8.3.23223580064

[B14] Cameroon National Institute of Statistics Cameroon third demographic and health survey 2004 3 http://www.measuredhs.com/pubs/pdf/SR107/SR107.pdf

[B15] Adu FD Adedeji AA Esan JS Odusanya OG Live viral vaccine potency: an index for assessing the cold chain system Public Health 1996 110 6 325 330 10.1016/S0033-3506(96)80003-5 8979747

[B16] WHO/UNICEF Review of national immunization coverage 1980–2005 2006 Cameroon: WHO http://www.unicef.org/publications/files/Immunization_Summary_2007.pdf

[B17] Bell KN Hogue CJ Manning C Kendal AP Risk factors for improper vaccine storage and handling in private provider offices Pediatrics 2001 107 6 E100 10.1542/peds.107.6.e100 11389298

[B18] Dipika MM Robertson J Garrison MM Newland S Carib N Freezing temperatures in the vaccine cold chain: a systematic literature review Vaccine 2007 25 20 3980 3986 10.1016/j.vaccine.2007.02.052 17382434

[B19] Turner N Laws A Roberts L Assessing the effectiveness of cold chain management for childhood vaccines J Prim Health Care 2011 3 4 278 282 22132380

[B20] Gazmararian JA Oster NV Green DC Schuessler L Howell K Davis J Krovisky M Warburton SW Vaccine storage practices in primary care physician offices: assessment and intervention Am J Prev Med 2002 23 4 246 253 10.1016/S0749-3797(02)00512-3 12406478

[B21] Mugharbel KM Al Wakeel SM Evaluation of the availability of cold chain tools and an assessment of health workers practice in Dammam J Family Community Med 2009 16 3 83 88 23012197 PMC3377047

[B22] Provincial vaccine coordinators national EPI. Cold chain and immunization operations manual guidelines for handling heat sensitive vaccines and pharmaceuticals 2003 South Africa: The department of health 31 39 http://www.savic.ac.za/backend/docs/Cold%20Chain%20Manual%202003.pdf

[B23] Dexiang C Debra K Opportunities and challenges of developing thermostable vaccines Expert Rev Vaccines 2009 8 5 547 557 10.1586/erv.09.20 19397412

[B24] Schlumberger M Mireux F Tchang SG Mboutbogol D Cheikh DO Hissein AA Youssouf BO Brahimi MM Gamatié Y Computerized temperature monitoring of the vaccine cold chain in a tropical climate Chad Med Trop 2011 71 3 264 266 21870554

